# Editorial: Advances and applications of cost-effective, high-throughput genotyping technologies for sustainable agriculture

**DOI:** 10.3389/fpls.2023.1335417

**Published:** 2023-12-08

**Authors:** Nisha Singh, Sapna Langyan, Vandna Rai

**Affiliations:** ^1^ Department of Bioinformatics, Gujarat Biotechnology University, Gandhinagar, India; ^2^ ICAR-National Bureau of Plant Genetic Resources, New Delhi, India; ^3^ ICAR-National Institute for Plant Biotechnology, New Delhi, India

**Keywords:** crop improvement, NGS, genomics, GWAS, high-throughput genotyping technologies, sustainable agriculture

Rapid breakthroughs in next-generation sequencing (NGS) technology have greatly aided crop improvement. It became a powerful tool for the production of genomic data, and various other integrated techniques have considerably expanded and deepened our understanding of living organisms’ molecular systems. NGS technologies bring novel tools and concepts that can enhance the precision and efficiency of plant breeding, such as the development of cost-effective, high throughput genotyping technologies and its various applications in sustainable agriculture. These technologies enable plant breeders to genotype a large number of samples in a short time span. It is used to implement genomic selection (GS) as a routine practice in breeding programs for fast-track development of superior crop breeds that are stress-resistant while still having a high nutritional value. In conventional plant breeding approaches, it takes a much longer time to create new, improved varieties because this relies on phenotypic selection and breeder experience. Moreover, complex quantitative traits have low heritability and are therefore difficult to select. Modern breeding procedures are advantageous because they are more reliable and efficient. Anand et al. explain that it is more feasible and sustainable to use cutting-edge technology to power agronomic development.

The application of genomic technologies such as genome-wide association studies (GWAS) and genomic prediction analysis (GPA) to durum wheat landrace resources paves the way for next-generation breeding programs to overcome the knowledge gap of these underexplored resources and to identify advantageous alleles lost in modern varieties. Therefore, to address the challenges of climate change, food, and nutritional security, Broccanello et al. emphasized the significance of durum wheat landraces as a valuable genetic resource for improving the sustainability of Mediterranean agroecosystems, with a focus on resilience to environmental stresses.

GS approaches have proven beneficial for assessing breeding values and phenotypic prediction using data from genome-wide molecular markers. To evaluate individual breeding value, the link between an individual genotype and a phenotype has been simulated using a variety of parametric approaches. To overcome the constraints of distinct parametric and nonparametric models, an integrated model for genomic prediction under additive and non-additive genetic architectures was developed by Budhlakoti et al. It can simultaneously handle both additive and epistatic effects by minimizing their error variance. Standard evaluation criteria such as prediction ability and error variance are used to evaluate the proposed integrated model. In the past few years, there has been an immense advancement in high-throughput phenotyping, phenomics, and computational biology, which has made it possible to explore such enormous datasets.

The significance of high-throughput sequencing and gene editing technologies for molecular breeding applications and trait-related genes in orchids is comprehensively explored by Song et al. This information will facilitate a scientific reference and theoretical basis for orchid genome studies. Yasir et al. used high-density single nucleotide polymorphism (SNP) arrays and DNA sequencing to disclose the bulk of the genotypic space for several crops, including cotton. The importance of GWAS and its various applications is emphasized. It was developed and first used in the field of human disease genetics. It connects the dots between a phenotype and its underlying genetics across population genomes and provides thoughtful insight into GWAS studies in cotton crop. Fiber yield and quality traits, GWAS status, prospects, and bottlenecks are discussed through case studies on both biotic and abiotic tolerance, a thought-provoking discussion. Exploring GWAS for dissecting competitive traits in major legume crops is well described by Susmitha et al. The study shed further light on advancements in biotechnology, sequencing, and several bioinformatics tools to estimate linkage disequilibrium (LD)–based associations. By computing genomic estimated breeding values (GEBVs), GWAS markers could be used for GS to forecast superior lines. However, it is yet to be employed in minor legumes where germplasm/population is available. Upadhyay et al. used a GWAS approach to evaluate the association for the acid phosphatase activity at various phosphorus levels in 280 mustard genotypes in two environmental conditions. A total set of 44 SNPs was identified that were significantly associated with two traits at three Pi (inorganic phospate) levels, for acid phosphatase (Apase) activity in the root (RApase) and leaf (LApase).These findings laid a solid foundation to improve the phosphorus use efficiency (PUE) of Indian mustard using marker-assisted selection in the future. This will redeem the crop by increasing the yield in the face of limited Pi reserves and deteriorating agro-environments.

Breeding parthenocarpic lines based on molecular markers is a faster and more efficient method. In cucumber, since selection may be based on genotypes rather than phenotypes, the identification of gynoecious traits and sex expression in cucurbitaceous vegetable crops has greatly aided the hybrid breeding program and has improved production, as reported by D. et al. The study of F2 progenies of PVGy-201 Pusa Do Mousami indicated that the genotype’s gynoecious sex expression is governed by a single recessive gene. The gynoecious sex expression is shown to be related to 1.31 Mb areas located on chromosome 1. A large number of variants were discovered in the QTL-region, which will aid in the precise mapping of the gynoecious characteristic.

Moreover, the parthenocarpic fruit set is a crucial characteristic in cucumbers, enabling large-scale, protected farming on a global scale. Devi et al. provide insight into genomic regions, closely associated markers, and possible candidate genes associated with parthenocarpy in Pusa Parthenocarpic Cucumber-6 (PPC-6), which will be instrumental for functional genomics study and better understanding of parthenocarpy in cucumber. The authors identified a major QTL, Parth6.1, associated with parthenocarpic fruit development in the slicing cucumber genotype PPC-6 using QTL-seq analysis in combination with conventional mapping using the F2:3 population.

The information gathered about genetic links and QTLs will be extremely beneficial for mango breeding and for increasing our understanding of the genetics of these traits. This insight was investigated by Srivastav et al. to enable mango breeders to develop trait-specific breeding methods. An 80K high density genic SNP chip array was used for genotyping to construct high resolution mapping of QTLs for fruit color and firmness in Amrapali/Sensation mango hybrids. This cross-produced 92 bi-parental offspring was utilized to create a high-density linkage map and to identify QTLs.

Systematic evaluation and cataloging of genetic variation at the morphological and phytochemical levels is particularly beneficial for effective conservation, utilization, and optimal genetic improvement of allelic and genotypic variability. There is a dearth of detailed information on genetic diversity in Kalmegh. Chaturvedi et al. identified 91 metabolic pathway-specific EST-SSR markers, of which, 32 EST-SSR primer pairs were chosen randomly for genetic diversity analysis within the six populations (ecotypes) of 24 kalmegh accessions (*Andrographis paniculata* (Burm. f.) Nee) for genetic improvement, germplasm conservation, and maximizing genetic gain.

In conclusion, this Research Topic focuses on recent advancements in NGS-related technologies, mainly the development of cost-effective, high-throughput genotyping platforms with a wide range of bioinformatics tools, and possible translational multi-omics applications in crop breeding programs for sustainable agriculture. We included a total of 13 publications in this Research Topic, which comprised of both original research and review papers. The Research Topic provides insights into cutting-edge research on various facets of emerging NGS, high-throughput genotyping technologies, GWAS, GS, and QTL mapping for identification and molecular breeding applications in a diverse array of crops such as cereals, legumes, and horticultural and medicinal plants. It is well proven that, over the last two decades, scientists have been able to uncover most of the genotypic space for diverse crops by using high-density SNP arrays and DNA sequencing. In this context, GWAS is considered a very powerful tool to identify key genes to unravel the mechanisms that will help devise efficient strategies for crop improvement and breeding programs. Furthermore, this will facilitate the investigation of the relationship between natural genetic variations and trait mapping in large populations. This Research Topic highlights the most recent genetic tools and statistical approaches ideal for the discovery of beneficial genes/alleles and associated with the most important traits in diverse crops for marker-assisted selection. The information gathered about genetic links and QTLs will be extremely beneficial for crop breeding and for increasing our understanding of the genetics of key agronomically important traits.

## Author contributions

NS: Conceptualization, Data curation, Formal Analysis, Investigation, Project administration, Resources, Supervision, Validation, Visualization, Writing – original draft, Writing – review & editing. SL: Formal Analysis, Visualization, Writing – review & editing. VR: Formal Analysis, Investigation, Validation, Visualization, Writing – review & editing.

